# Moving from one to many: insights from the growing list of pleiotropic cancer risk genes

**DOI:** 10.1038/s41416-019-0475-9

**Published:** 2019-05-21

**Authors:** Stephanie A. Bien, Ulrike Peters

**Affiliations:** 1 Division of Public Health Sciences, Fred Hutchinson Cancer Research Center, Seattle, WA 98109 USA; 2 Department of Epidemiology, School of Public Health, University of Washington, Seattle, WA 98109 USA

**Keywords:** Cancer genetics, Cancer genetics

## Abstract

Pleiotropy, a phenomenon in which a single gene affects multiple phenotypes, is becoming very common among different cancer types and cancer-related phenotypes, such as those in hormonal, cardiometabolic and inflammatory/immune conditions. The discovery of pleiotropic associations can improve our understanding of cancer and help to target investigation of genes with greater clinical relevance.

## Main

Closely following heart disease, cancer is the second leading cause of death in westernised populations. The complex biology of cancer is underscored by the discovery of more than 1000 low-penetrance cancer risk variants.^1^ Estimates of shared genetic heritability between different cancer types have shown statistically significant correlations, with estimates as high as *r*_g_ = 0.55 for pancreatic and colorectal cancer.^2^ These estimates are consistent with expectations of extensive pleiotropy among polygenic traits.^3^ Accordingly, it is not surprising that genome-wide association studies (GWAS) have highlighted many commonalities in genetic risk and overlap in key pathways across cancer types. Some of the most prominent pleiotropic genes include *MYC*, *TERT* and *HNF1B*, all of which are linked to a growing number of cancer types. Recent cross-cancer GWAS have identified seven new pleiotropic genes that were not previously discovered by single-trait analysis, further demonstrating that this approach can power new discoveries.^4^

**Fig. 1:**
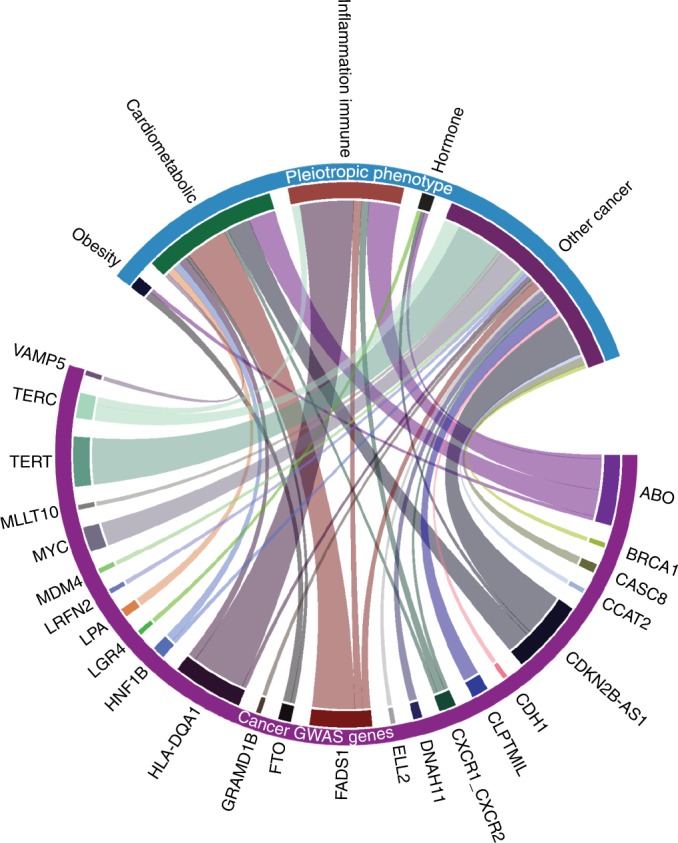
Examples of pleiotropic genes near a GWAS-identified cancer locus associated with another cancer and/or phenotype in a relevant condition. Each gene represents a pleiotropic locus that is associated with multiple cancer types and/or one cancer type, and cancer-related traits and biomarkers. Connections in this chord diagram indicate that variants in or near respective genes associate with both a cancer type and one or more phenotypes within the linked conditions. The width of the chord corresponds to the number of phenotypes within the respective area; for example, the widest chord between *TERT* and Other cancer represents association between the *TERT* locus and 12 different cancers

Despite the fact that pleiotropy is pervasive throughout the human genome, investigations to characterise the shared genetic basis of common cancers and other cancer-related phenotypes remain limited, but the plethora of pleiotropy findings revealed through ad hoc analyses (Fig. 1) suggest that many additional shared genetic risk genes exist. Here we highlight key examples of the insights gained from comprehensive and systematic cross-cancer GWAS analyses. Pleiotropic discoveries can (1) identify shared biologic pathways and prioritise probable causal relationships, (2) reveal unexpected links between phenotypes and aid in aetiological disease classification, (3) test key assumptions for Mendelian randomisation studies, (4) inform repurposing of drugs and predict adverse drug reactions, and (5) increase the statistical power.

## Shared biological pathways and unexpected phenotypic links

Pleiotropy has for long been described in monogenic diseases because high-penetrance mutations often cause a constellation of seemingly unrelated clinical features.^5^ As an example, PTEN hamartoma tumour syndrome (PHTS), which is caused by mutations in *PTEN*, predisposes to multiple cancers. PHTS is characterised by multiple hamartomas – benign tumour-like malformations comprising an abnormal mixture of cells and tissues – that can arise in any organ. Although PTEN is a tumour suppressor, it is also involved in non-canonical pathways, meaning that individuals with PHTS can also suffer from severe disfigurement and intellectual disability.^6^ This is referred to as biological pleiotropy (e.g. cancer ← G_PTEN_ → intellectual disability). By contrast, pleiotropic associations can also arise when one phenotype influences another. Take, for instance, *CHRNA5*, a gene that associates with lung cancer, chronic obstructive pulmonary disease (COPD) and smoking behaviours. Associations with lung cancer could be due to the profound effects of *CHRNA5* variants on smoking intensity, either directly or indirectly through effects on COPD, in a phenomenon referred to as mediated pleiotropy (G_*CHRNA*_ → smoking → COPD → lung cancer). Systematic analysis of possible pathways between G_*CHRNA*_ and lung cancer risk suggests that both direct and mediated effects contribute, with approximately 40% attributed to smoking (directly or through COPD).^7^ Systematic investigations can provide critical new insight into shared disease mechanisms, causal relationships or novel biological pathways. However, little attention has been given to the study of pleiotropy in complex phenotypes, as opposed to in Mendelian disease. GWAS have provided ample evidence that complex traits are highly polygenic, which has led to the establishment of very large case-control studies and encouraged super-consortia usually focusing on a single disease. The rapid discovery of variant associations by these ‘disease-specific’ consortia has, however, detracted from efforts to find pleiotropic key regulator genes with far-reaching aetiological influences, and hindered the ability to readily perform cross-trait analyses.

GWAS have identified many genetic risk factors that are shared between cancers and other related phenotypes, such as cardiometabolic (*CDKN2B-AS1*, *HNF1B*), inflammatory/immune (*CDKN1B*, *FADS1)*, obesity (*FTO*), or hormonal (*LGR5*) conditions. Some of these associations initially seemed rather surprising, such as the positive link between prostate cancer and *HNF1B*, which also showed a reduced risk for type 2 diabetes; however, this result is consistent with the observation that individuals with type 2 diabetes are at decreased risk for prostate cancer^8^ – an unexpected association that had previously been given limited attention.

## Mendelian randomisation

The number of publications involving Mendelian randomisation studies has rapidly increased as of late; most likely, this reflects their purported ability to estimate causal effects in observational settings. In this capacity, Mendelian randomisation has been proposed as a pharmacovigilance and drug-repurposing tool to help identify treatment targets and to prioritise (or deprioritise) major investments in randomised controlled trials (RCTs). In this setting, Mendelian randomisation involves finding genetic variants associated with a modifiable target (e.g. plasma selenium and dietary supplementation), and then testing the association between those variants and the outcome (e.g. prostate cancer).^9^ However, the absence of pleiotropy is a core assumption that underlies Mendelian randomisation studies, and violation of this assumption can cause severe bias. For example, if the genetic variants used as a proxy for an intended target are associated with decreasing cancer risk through an alternative pathway, the drug or supplement in question could be completely ineffective, or even harmful, despite support from Mendelian randomisation. The extent of pleiotropy among complex traits and diseases is only beginning to be appreciated. As we typically only assess pleiotropy in the context of variants that have already been reported, more comprehensive cross-trait studies are needed before we continue to replace true RCTs with an imperfect statistical approach.

## Drug repurposing

It is estimated that the success rate for drug development could be doubled if the selection of drug targets is supported by evidence from human genetic studies.^10^ The examples above demonstrate how the discovery of pleiotropic associations can improve RCT design, by screening for subtypes and adverse drug reactions. Identifying pleiotropy can also help to repurpose existing drugs, avoiding de novo development and further predict adverse drug events, thereby redirecting the efforts to more promising targets before the inception of an RCT.

The extent of pleiotropy between cancer loci and other seemingly disparate diseases and traits presented in Fig. 1 are intriguing. So far, few studies have performed genome-wide pleiotropic analyses between cancer traits and other complex diseases. Thus, because the results in Fig. 1 come from the comparison of results from single-trait GWAS, it is likely that the extent of pleiotropy is vastly underestimated given that pleiotropic analyses increase the statistical power for new discoveries. Therefore, the new era of GWAS should move away from the narrowly focused cataloguing of genotype and single-phenotype associations, and take into account comprehensive cross-trait analyses if we wish to fully realise the goals of precision medicine.

## Data Availability

Not applicable.
